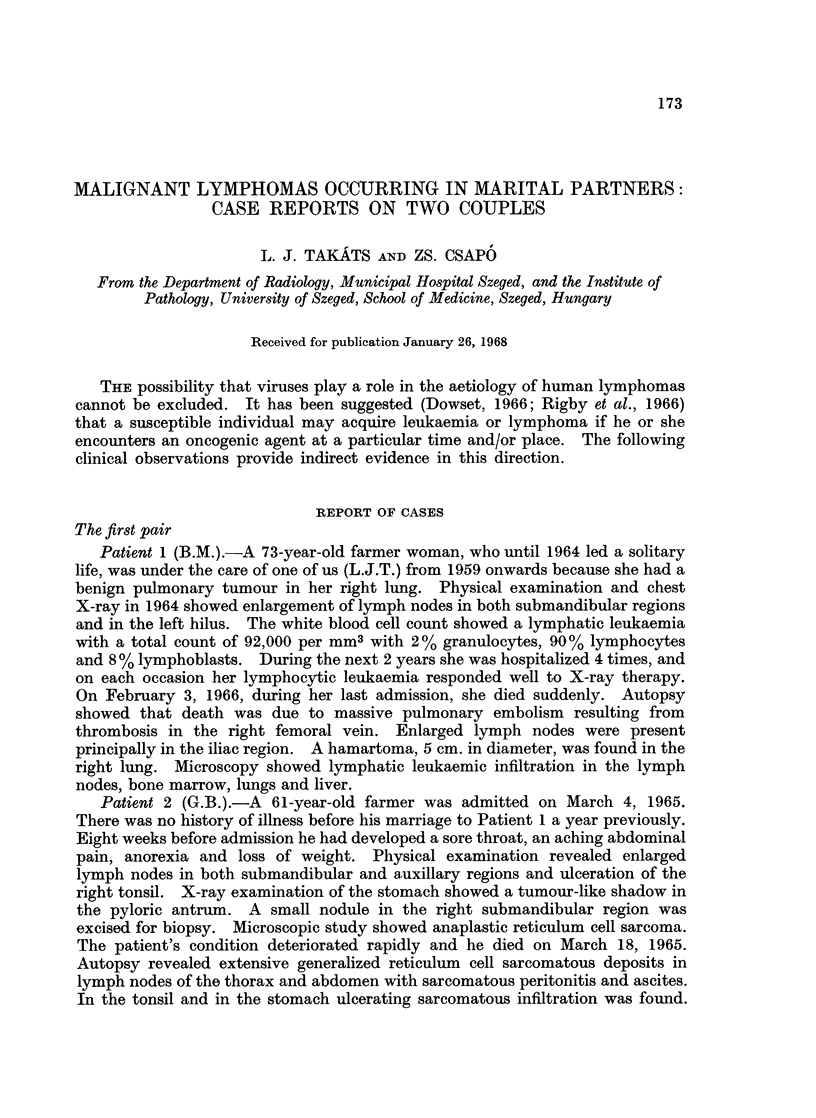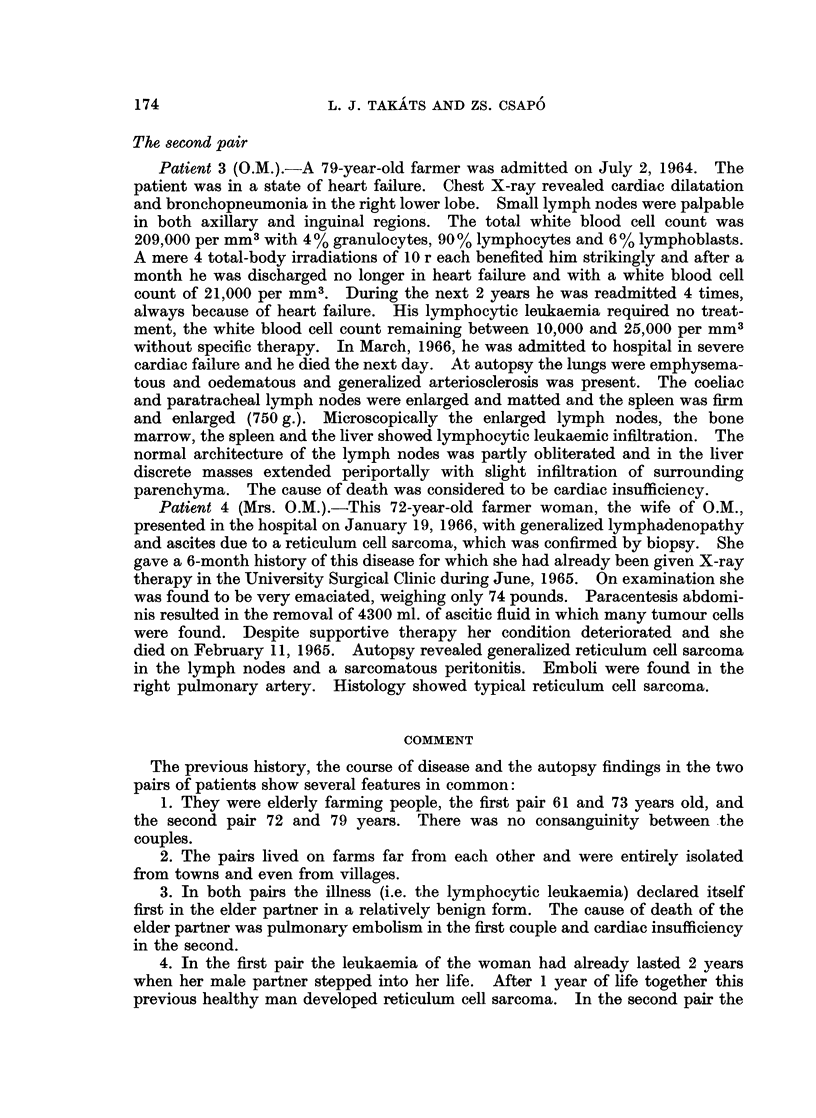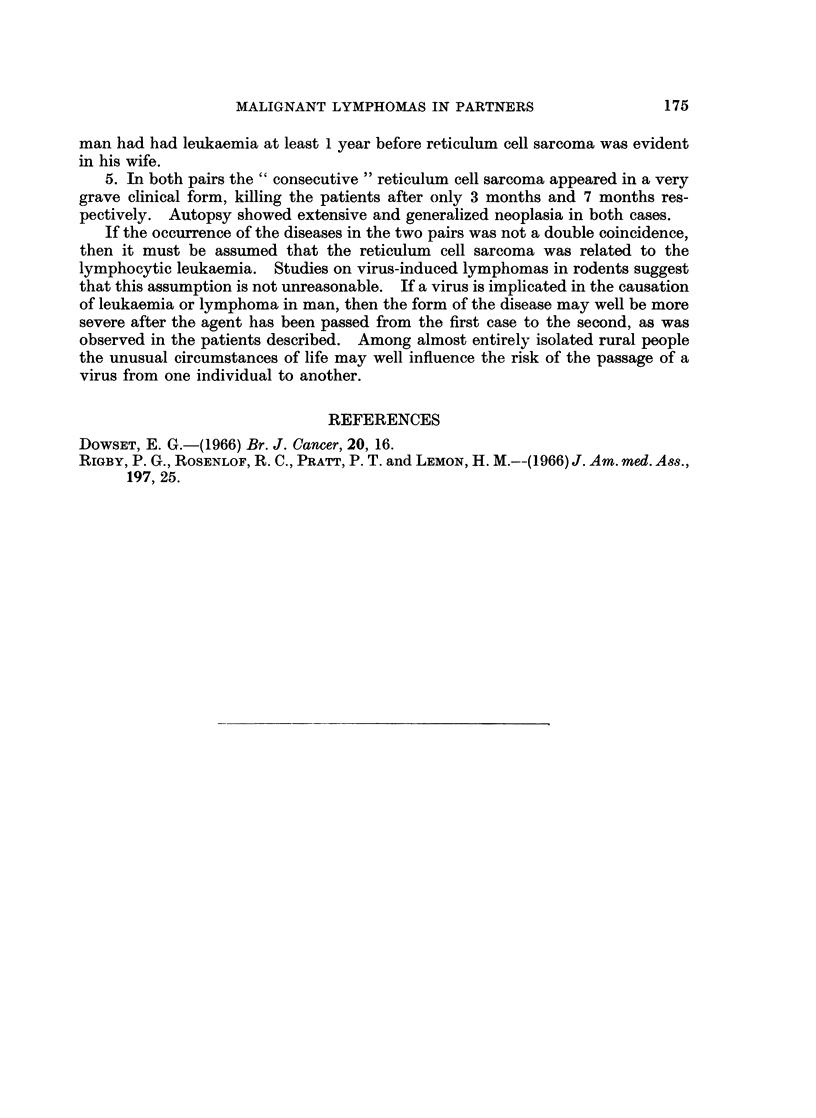# Malignant lymphomas occurring in marital partners: case reports on two couples.

**DOI:** 10.1038/bjc.1968.24

**Published:** 1968-06

**Authors:** L. J. Takáts, Z. Csapó


### MALIGNANT LYMPHOMAS OCCURRING IN MARITAL PARTNERS: CASE REPORTS ON TWO COUPLES

L. J. TAKÁTS AND ZS. CSAPÓ

*From the Department of Radiology, Municipal Hospital Szeged, and the Institute of Pathology, University of Szeged, School of Medicine, Szeged, Hungary*

Received for publication January 26, 1968

THE possibility that viruses play a role in the aetiology of human lymphomas cannot be excluded. It has been suggested (Dowset, 1966; Rigby *et al.*, 1966) that a susceptible individual may acquire leukaemia or lymphoma if he or she encounters an oncogenic agent at a particular time and/or place. The following clinical observations provide indirect evidence in this direction.

### REPORT OF CASES

### *The first pair*

*Patient* 1 (B.M.).—A 73-year-old farmer woman, who until 1964 led a solitary life, was under the care of one of us (L.J.T.) from 1959 onwards because she had a benign pulmonary tumour in her right lung. Physical examination and chest X-ray in 1964 showed enlargement of lymph nodes in both submandibular regions and in the left hilus. The white blood cell count showed a lymphatic leukaemia with a total count of 92,000 per $mm^3$ with 2% granulocytes, 90% lymphocytes and 8% lymphoblasts. During the next 2 years she was hospitalized 4 times, and on each occasion her lymphocytic leukaemia responded well to X-ray therapy. On February 3, 1966, during her last admission, she died suddenly. Autopsy showed that death was due to massive pulmonary embolism resulting from thrombosis in the right femoral vein. Enlarged lymph nodes were present principally in the iliac region. A hamartoma, 5 cm. in diameter, was found in the right lung. Microscopy showed lymphatic leukaemic infiltration in the lymph nodes, bone marrow, lungs and liver.

*Patient* 2 (G.B.).—A 61-year-old farmer was admitted on March 4, 1965. There was no history of illness before his marriage to Patient 1 a year previously. Eight weeks before admission he had developed a sore throat, an aching abdominal pain, anorexia and loss of weight. Physical examination revealed enlarged lymph nodes in both submandibular and auxillary regions and ulceration of the right tonsil. X-ray examination of the stomach showed a tumour-like shadow in the pyloric antrum. A small nodule in the right submandibular region was excised for biopsy. Microscopic study showed anaplastic reticulum cell sarcoma. The patient's condition deteriorated rapidly and he died on March 18, 1965. Autopsy revealed extensive generalized reticulum cell sarcomatous deposits in lymph nodes of the thorax and abdomen with sarcomatous peritonitis and ascites. In the tonsil and in the stomach ulcerating sarcomatous infiltration was found.

*The second pair*

*Patient* 3 (O.M.).—A 79-year-old farmer was admitted on July 2, 1964. The patient was in a state of heart failure. Chest X-ray revealed cardiac dilatation and bronchopneumonia in the right lower lobe. Small lymph nodes were palpable in both axillary and inguinal regions. The total white blood cell count was 209,000 per $mm^3$ with 4% granulocytes, 90% lymphocytes and 6% lymphoblasts. A mere 4 total-body irradiations of 10 r each benefited him strikingly and after a month he was discharged no longer in heart failure and with a white blood cell count of 21,000 per $mm^3$. During the next 2 years he was readmitted 4 times, always because of heart failure. His lymphocytic leukaemia required no treatment, the white blood cell count remaining between 10,000 and 25,000 per $mm^3$ without specific therapy. In March, 1966, he was admitted to hospital in severe cardiac failure and he died the next day. At autopsy the lungs were emphysematous and oedematous and generalized arteriosclerosis was present. The coeliac and paratracheal lymph nodes were enlarged and matted and the spleen was firm and enlarged (750 g.). Microscopically the enlarged lymph nodes, the bone marrow, the spleen and the liver showed lymphocytic leukaemic infiltration. The normal architecture of the lymph nodes was partly obliterated and in the liver discrete masses extended periportally with slight infiltration of surrounding parenchyma. The cause of death was considered to be cardiac insufficiency.

*Patient* 4 (Mrs. O.M.).—This 72-year-old farmer woman, the wife of O.M., presented in the hospital on January 19, 1966, with generalized lymphadenopathy and ascites due to a reticulum cell sarcoma, which was confirmed by biopsy. She gave a 6-month history of this disease for which she had already been given X-ray therapy in the University Surgical Clinic during June, 1965. On examination she was found to be very emaciated, weighing only 74 pounds. Paracentesis abdominis resulted in the removal of 4300 ml. of ascitic fluid in which many tumour cells were found. Despite supportive therapy her condition deteriorated and she died on February 11, 1965. Autopsy revealed generalized reticulum cell sarcoma in the lymph nodes and a sarcomatous peritonitis. Emboli were found in the right pulmonary artery. Histology showed typical reticulum cell sarcoma.

### COMMENT

The previous history, the course of disease and the autopsy findings in the two pairs of patients show several features in common:

1. They were elderly farming people, the first pair 61 and 73 years old, and the second pair 72 and 79 years. There was no consanguinity between the couples.
2. The pairs lived on farms far from each other and were entirely isolated from towns and even from villages.
3. In both pairs the illness (i.e. the lymphocytic leukaemia) declared itself first in the elder partner in a relatively benign form. The cause of death of the elder partner was pulmonary embolism in the first couple and cardiac insufficiency in the second.
4. In the first pair the leukaemia of the woman had already lasted 2 years when her male partner stepped into her life. After 1 year of life together this previous healthy man developed reticulum cell sarcoma. In the second pair the man had had leukaemia at least 1 year before reticulum cell sarcoma was evident in his wife.

5. In both pairs the " consecutive " reticulum cell sarcoma appeared in a very grave clinical form, killing the patients after only 3 months and 7 months respectively. Autopsy showed extensive and generalized neoplasia in both cases.

If the occurrence of the diseases in the two pairs was not a double coincidence, then it must be assumed that the reticulum cell sarcoma was related to the lymphocytic leukaemia. Studies on virus-induced lymphomas in rodents suggest that this assumption is not unreasonable. If a virus is implicated in the causation of leukaemia or lymphoma in man, then the form of the disease may well be more severe after the agent has been passed from the first case to the second, as was observed in the patients described. Among almost entirely isolated rural people the unusual circumstances of life may well influence the risk of the passage of a virus from one individual to another.

### REFERENCES

Dowset, E. G.—(1966) *Br. J. Cancer*, **20**, 16.
Rigby, P. G., Rosenlof, R. C., Pratt, P. T. and Lemon, H. M.—(1966) *J. Am. med. Ass.*, **197**, 25.